# Permafrost Immunity

**DOI:** 10.6026/97320630018734

**Published:** 2022-09-30

**Authors:** Francesco Chiappelli, Jaden Penhaskashi

**Affiliations:** 1 Dental Group of Sherman Oaks, CA 91403 (www.oliviacajulisdds.com); 2 West Valley Dental Implant Center, Encino, CA 91316 (minimallyinvasiveperio.com)

**Keywords:** Climate change, Global warming, Permafrost, Immune allostasis, Cellular immunity, Humoral immunity, Exhausted T cells, Cytokine Storm, Virus-Induced Autoimmunity, Oral health

## Abstract

Thawing permafrost is a serious and worrisome threat to the environment, because it releases trapped heavy metals and greenhouse gasses. Thawing permafrost is also a health threat because, in addition to releasing these noxious gasses, thawing permafrost may free novel and undiscovered antibiotic-resistant bacteria, viruses, fungi and parasites among a plethora of dormant pathogens. Our immune system is ill-prepared to counter these challenges, and will require significant adaptation, or allostasis, which can be subsumed under the generic term of permafrost immunity. Since most of the most gravely threatening pathogens released by thawing permafrost are likely to penetrate the organism through the oral cavity, permafrost immunity may first be identified in the oral mucosa.

## Background:

Ever since the onset of the Industrial Revolution in the 1850s, our planet has suffered a mounting onslaught of pollution, which has progressively altered the fundamental cleanliness of its air, water and soil, and favored the rise of new and ancient health threats [1]. Rising levels of greenhouse gases trapped in the atmosphere has led to increased air temperatures, which has translated into warmer seas and oceans. The process, unabated over decades, has led to the inexorable changes in climate patterns we experience today across the planet, from pole to pole and at every latitude.

Global warming also causes melting of glaciers and eternal snows at higher altitudes, and the release of large sheets of polar ice into the oceans. On land, extended areas of permafrost are thawing [2]. Permafrost is generally defined as the ground where soil temperature remains at or below 0°C for at least two consecutive years. However, permafrost actually consists of an upper active layer of frozen soil that may, or may not freeze and thaw seasonally, and a second deeper layer of soil that is largely stable and can remain frozen for centuries. The ratio of active and stable permafrost varies geographically. The rising concern over the past decade is the impact of climate change and the possibility of subsequent permafrost thaw in turn promoting ancient and novel microbial activity captured in permafrost ice [3].

Certain of these pathogens may be serious public health threats, including ancient lethal variants of Yersinia pestis, the plague-causing bacterium, influenza virus (e.g., Spanish Flu 1919 pandemic), and anthrax [3-9]. Fragments of DNA and RNA from diseases such as smallpox, bubonic plague, as well as the 1918 influenza virus can be recovered from human and animal remains harbored in peri-Arctic and peri-Antarctic areas [10].

Groundbreaking and improved DNA sequencing technologies allow a glimpse into ancient DNA of pathogens long entrapped in permafrost. Bacteria, viruses, and fungi, including from periodontal and more generally oral pathogens, can be recovered from thawing permafrost, and their genomes compared to their extant counterparts. Permafrost microbial history is furthermore enriched by ancient pathogens extracted from specimens of mummified bones and dental pulp released from thawing permafrost [11].

A recent review identified at least ten bacteria, from *Yersinia pestis, Bartonella quintana, Salmonella enterica serovar Typhi, Salmonella enterica serovar Paratyphi C, Mycobacterium leprae, Mycobacterium tuberculosis, Rickettsia prowazeki, Staphylococcus aureus,* and *Borrelia recurrentis,* to *Bartonella henselae,* and at least one virus of the *Anelloviridae* family can be recovered from ancient permafrost dental pulp specimens [12]. Taken together, the evidence to date suggests that health-threatening novel and ancient pathogens are released from thawing permafrost. In addition, pathogens from the oral cavity are also being recovered from melting glaciers and permafrost, confirming the urgency to better understand permafrost immunity.

## Discussion:

(2017 words) Immunity is best defined as the physiological system that protects from pathogens that could otherwise be harmful or even lethal. Immunity is proffered by the immune system, the set of finely concerted and integrated biochemical and cellular processes and regulated events and pathways, which act together in perfect orchestrated unison. Immune processes and events can be either adaptive and specific in nature, or innate and largely nonspecific. Permafrost immunity encompasses both the native and adaptive immune responses to pathogens released by thawing permafrost.

As one principal aspect of the physiological processes that regulate and restore health following illnesses and infectious diseases, immunity is itself driven to homeostasis. Physical, psycho-emotional, and physio-pathological stress drive a loss in homeostasis with potentially profound sequelae, which can involve significant immune suppression and impairment. The natural process of recovering homeostasis is termed allostasis [13], which encompasses the set of disparate, yet concerted events that involve whole-brain and whole-body regulation, rather than simple local feedback. In brief, allostasis is the response to changing circumstances, diverse environments, and stressful challenges coordinated by the organism's central and peripheral nervous, neuro-endocrine and psycho neuro-endocrine-immune systems [14]. Permafrost immunity can be defined, in short, as the concerted homeostatic adaptive regulation of immunity in general, and specific immune surveillance mechanisms in particular, to given allostatic threats provoked by the pathogens released by melting ice.

There are two distinct types of allostatic threats: that which is a temporary and reversible load, and that which is de facto - a chronic, constant, irreversible, or even consistently growing overload. As much as it is possible to attain allostasis, stability through change of state, to reversible and temporary allostatic threats (Type I allostasis), allostasis to irreversible chronic threats is realistically and factually unreachable (Type II allostasis) [14, 15]. For example, the pain and discomfort produced by aphthous stomatitis (i.e., canker sore) is reversible, as such as a prime example of Type I allostasis in the oral cavity; by contrast, the pain and suffering of the oral sores produced by the incurable, chronic and progressively exacerbating Oral Lichen Planus exemplifies a Type II allostasis [15].

In the context of immunity, immune allostasis is critical for maintaining a strong and efficient resistance against pathogens [16], as well as cancer immune surveillance [17]. Since allostatic load and overload are associated with poorer health outcomes, it follows that assessment of allostatic load, and characterization of Type I versus Type II allostatic load provide support to the understanding of the psychosocial, psychoemotional and psychoneuroendocrine determinants of immunity in particular, and health in general[15-17].

Allostasis and allostatic load are foundational to the science of psychoneuroimmunology, the study of the intertwined interdependence among psychosocial and psychoemotional behaviors and cognitive responses, the central and peripheral nervous regulation of endocrine signals, and cellular and humoral immune surveillance processes. More generally, the existence of bidirectional communication pathways between the brain and the immune system and the implications of this network for perceived allostastic load differential (i.e., reversible Type I vs. inescapable and irreversible Type II) dictate and determine anti-viral, anti-bacterial and anti-tumor immune efficiency [18] to an extent that can be predicted and modeled in artificial intelligence algorithms [19].

In an ever-magnifying set of environmental threats, some perceived by the organism as reversible while others as chronic and consistently rising, the specific pattern of psychobiological labor each individual organism undergoes to regain a balanced state of homeostasis in response to climate change and global warming can be defined as the allostasiome [20]. The allostasiome inexorably involves a variety of physiological processes, including immune surveillance pathways and processes. To be clear, it is probable and even likely that immunity will become increasingly impaired as our global climate progressively worsens. The concerted literature already points to a more comprehensive picture of the environmental causes of autoimmune diseases [21]. Indeed, the current rises in autoimmune (e.g., psoriasis) and allergic diseases (e.g., asthma) may be a direct consequence of the noxious effects of climate change and global warming on the allostasiome.

Certain viruses, which we considered limited to the tropics, have aggressively invaded higher latitudes engendering dangerous pandemics (i.e., SARS-CoV2 responsible for CoViD-19, 22) and epidemics of concern (e.g., monkeypox virus). While they do not originate from the thawing permafrost per se, these pathogens exemplify the broad nature of the threat to the immune system resulting from climate change and global warming. It is possible and even likely that the novel and ancient pathogens released the melting permafrost over the next decades will challenge immune surveillance mechanisms to an even greater extent. Case in point, challenge by the SARS-CoV2 virus leads to immune suppression across a variety of domains, from an increase in the absolute number and relative percentage of T cells that express manifested programmed cell death marker-1 (PD-1, CD279) and the T cell immunoglobulin and mucin domain 3 (Tim-3), and which together are markers of "exhausted" T cells [23], to deregulated cytokine "storm" [23,24] and impaired B cell responses manifested as auto-immunity [24]. Of course, these are but a few among the myriad of alterations in natural and adaptive immunity that together signify broad-based immune suppression [23-25], and which are becoming characteristics of the current pandemic (SARS-CoV2) and epidemic situations (Monkeypox virus).

Various permafrost viruses and bacteria have been identified at different geographical locations (Table 1 - see PDF). In brief, and to highlight those listed in the table, in Stordalen Mire, the subarctic region in northernmost Sweden, about 5 miles east of the town of Abisko close to Lake Torneträsk, the discontinuous permafrost evinces a large 25ha palsas area; that is, an area that consists of relatively tall peat mounds up to 12 m high and 150 m in diameter with a permanently frozen peat and mineral soil core. The active palsas permafrost of the Stordalen Mire has revealed the presence of Variola virus of the 170-250 kb DNA Orthopoxvirus genus. This genus consists of two principal families, of which the Poxviridae is the largest and causes pox-related pathologies (i.e., smallpox [=variola], cowpox, horsepox, camelpox, and monkeypox). The Variola virus was thought to have been eradicated globally by 1977 through the use of Vaccinia virus vaccine. The current rise in Monkeypox virus infection worldwide, and the emergence of permafrost Variola virus, however, is important threats to human health worldwide. The discovery of a know Orthopoxvirus in the Sweden permafrost, came to confirm earlier reports of another pox virus, the novel Alaskapox virus, uncovered in the thawing permafrost of the Fairbanks North Star Borough. The Alaskapox virus led to human fatalities [31].

The active palsas permafrost of the Stordalen Mire also revealed the presence of tailed bacteriophages, the group I virus of double stranded DNA Order of Caudovirales that includes the Myoviridae, the family of the Podoviridae. While representatives of the Caudovirales Order share similarities, such as being lytic to the bacteria they bind to, they also have characteristically distinctive features. Case in point, Podoviridae duplicate cytoplasmically, while Myroviridae are typically directly lytic. In general, the representatives of the Order of Caudoviradae are considered potential candidates for therapy against bacterial diseases, and thus may be considered beneficial rather than threats to human health.

In the lowlands of northeastern Siberia, the Kolyma River was historically frozen to depths of several meters for close to 250 days yearly. Recent measurements show a thinner ice sheet 4-6 months of the year, indicating thawing permafrost along the River banks. The ancient amoebae-infecting giant double-stranded circular DNA virus, Pithovirus sibericum, emerged from the Kolyma ice, as well as the spherical group I double stranded DNA Mollivirus sibericum. While neither is apparently harmful to humans, the latter may be considered a threat since it, and other members of its family, can be potentially noxious to human health because of their ability to sequester the host's ribosomal proteins in its vision.

In the Yamalo-Nenets autonomous Region of Okrig in Russian Siberia, in the northern polar Ural (about 70°N and 70°E), the permafrost of the arctic tundra and taiga have released alarming levels of what is commonly known to cause anthrax, a lethal bacterial infection brought about by the spores released by the Gram-positive 3-5 µm long rod-shaped Bacillus anthracis. Due to its high level of risk to human health, an aggressive program of vaccine development against B. anthracis has been deployed, leading to the development of the Anthrax vaccine adsorbed (AVA), directed to protect against cutaneous and inhalation anthrax. In most instances, however, the Anthrax vaccine AVA is, at this time, restricted for preventive use only in at-risk adults prior to anticipated exposure. It is timely and critical to approve generalized utilization of the vaccine for use after exposure as well. Post-exposure to the infectious agent, the only effective intervention remains at this time treatment for anthrax with β-lactam antibiotics (e.g., penicillin) [32]. Penicillin-resistant B. anthracis is treated with fluoroquinolones (e.g., ciprofloxacin) or tetracycline antibiotics (e.g., doxycycline).

In the Siberian permafrost along the Unga-Baga-Olonso stream (59° 31'49.11 N, longitude and 123° 0'15.58" E, at about 550 m elevation) near the city of Olyokminsk, Siberia, were found emerging along the active permafrost several pathogens among which the Gram-negative, oxidase- and catalase-positive Achromobacter insolitus of the Alcaligenaceae family typically pathogenic to humans, the rod-shaped metabacillus, Bacillus idriensis of the Gram-positive, heterotrophic Bacillaceae family that also cause disease of varying degree of severity in humans, and of the heterotrophic, Brevundimonas aurantiaca, a Gram-negative, non-fermenting, aerobic bacillus of the Caulobacteraceae family, which is rarely, if ever, recovered from clinical samples despite causing bacterimia of diverse severity [33]. In addition, samples at that site also revealed two Gram-positive bacteria innocuous to humans for all intents and purposes: the aerobic bacterium isolated from spoiled melon and other Cucurbitaceae fruits, Janibacter melonis of the Intra sporangiaceae actinomycete family, and the soil dwelling bacterium of the Micrococcaceae family Kocuria rhizophila.

To be clear, this list is neither complete not exhaustive. It simply seeks to exemplify the variety of viral and bacterial pathogens emerging from thawing planetary permafrost. The permafrost microbiotome - the uncovered ever evolving in complexity microbiology, virology, bacteriology and parasitology of the thawing permafrost-evinces the prevalence of deterministic or purely stochastic processes that can hypothetically predict whether we will continue to be well-suited to survive and to thrive in changing environmental conditions that accompany permafrost thaw. The predictive model can be conceived as an expansion of the AI approach we proposed to describe viral immune surveillance [19], because current and predicted conditions of climate change and global warming are expected to influence directly the microbial communities - the permafrost microbiotome - that result from the decomposition of formerly frozen organic matter, and that inexorably contribute further to uncertainty in permafrost-climate feedbacks [34], and therefore to challenge the anti-viral and and anti-bacterial immune surveillance processes in new and unforeseeable ways.

In brief, it is possible and even likely that novel and ancient pathogens released from thawing permafrost could even more seriously perturb immune regulatory mechanisms and the immune allostasiome in general. It follows that together; these alterations will define and characterize a new cadre of immunity, identifiable as permafrost immunity. To ensure our survival, our immune system facing permafrost-released pathogens will be challenged to favor one over another biochemical pathway and molecular process, one over another lymphocyte population, or one over another cytokine profile. Immune surveillance processes will have to adapt to optimize the neutralization of permafrost pathogens, an allostatic adaptation of immunity that is referred to as "permafrost immunity". Pathogens often first enter our organism through the nasal and the oral cavity. Therefore, it should be expected that the first of signs and symptoms of permafrost pathogens will be evinced at those anatomical sites. It follows that permafrost immunity will become evident the soonest in the mucosal immune compartment of the oral cavity and upper respiratory track.

## Conclusion:

As global warming and climate change prevail, glaciers on our planet's highest peaks and polar, peri-polar and sub-polar permafrosts are melting. As this process unfolds, noxious greenhouse gases, such as methane and carbon dioxide are released, rising global temperatures. In turn, novel and ancient pathogens, from viruses to bacteria and other parasites are freed from the eternal ice. Our immune system is increasingly called to mount efficient defenses against these biological threats, and to attain new states of homeostatic balance. These allostatic changes include certain immune cell populations and their function and cytokine profiles. The physiological adaptation of the immune system to the demands of permafrost pathogens discussed in this paper as permafrost immunity is akin conceptually to the "molding" of immunity, the modulation of mucosal and systemic immunity by the intestinal mycobiome in health and disease [35]. Permafrost-induced up-molding of immunity is anticipated to be pervasive and to involve every aspect of the immune system, from innate to adaptive immunity, from cellular to humoral immunity, and at the cellular level from T cell to B cell immunity. It is also argued that, because many, perhaps even most of the most gravely threatening pathogens (e.g., influenza virus, corona virus, E. coli bacteria) penetrate the organism through the oral cavity, it is in the mucosa of the mouth and of the upper respiratory tract that permafrost immunity will first become evident. In conclusion, it is timely and critical to begin proactive systematic exploration of the new frontiers of permafrost immunity to be on the alert when novel and ancient diseases will begin to seriously impact human health, consequential to the pathogens released by the thawing permafrost.
